# Multilevel thoracic ossification of ligamentum flavum coexisted with/without lumbar spinal stenosis: staged surgical strategy and clinical outcomes

**DOI:** 10.1186/s12891-015-0672-5

**Published:** 2015-08-19

**Authors:** Wen-jing Li, Shi-gong Guo, Zhi-jian Sun, Yu Zhao

**Affiliations:** Department of Orthopaedics, Peking Union Medical College Hospital, Chinese Academy of Medical Sciences and Peking Union Medical College, Dong Cheng District Shuai Fu Yuan No.1, Beijing, 100730 China; Department of Trauma & Orthopaedic Surgery, Lister Hospital, Stevenage, UK

## Abstract

**Background:**

Thoracic ossification of ligamentum flavum (TOLF) is a progressively disabling disease. Isolated or continuous TOLF has been frequently reported in literature, however there are very few reports of multilevel or non-continuous TOLF. The purpose of the study was to discuss the surgical strategy of multilevel TOLF and evaluate safety and efficacy of a two-stage operation regimen.

**Methods:**

From October 2007 to May 2014, eleven patients (4 males, 7 females) that underwent two-stage surgery for multilevel spinal stenosis were retrospectively reviewed. The follow-up period lasted at least 12 months. Demographic data, radiological findings as well as operative data were collected. Postoperative functional outcomes evaluated by the modified Japanese Orthopedic Association score (mJOA) and complications were analyzed.

**Results:**

The patients ranged in age from 30 to 65 years (average, 50.2 ± 11.8 years), and comprised 4 men and 7 women. All patients exhibited significant improvements in neurological deficits. The mJOA score improved from a mean of 3.5 ± 2.2 preoperatively to 4.6 ± 2.3 before second-stage surgery and to 7.5 ± 2.2 at final follow-up. The improvement was statistically significant in the average mJOA improvement rate at final follow-up. No staging-related complications were noted in this study.

**Conclusions:**

Staged surgery can effectively achieve neurological functional recovery in patients with multi-segment spinal stenosis in thoracic and lumbar regions, with favorable efficacy and safety. Yet, slight neurological deterioration was observed during the intervals of these two index surgeries.

## Background

Thoracic spinal stenosis (TSS) is a rare disease found almost exclusively in East Asians [1]. Ossification of ligamentum flavum (OLF) was recognized as a major cause of acquired TSS from the use of diagnostic imaging, such as computed tomography (CT) and magnetic resonance imaging (MRI) [1–3]. TOLF is a slowly progressive disease that can be readily diagnosed. However, once symptomatic, progresses rapidly and surgical management is unavoidable. Delayed time to surgery has been reported to be the key contributory factor in unfavorable surgical outcome in TOLF [2, 4]. Owing to some unclear causative factors, TOLF frequently coexists with other spinal disorders, such as lumbar spinal stenosis (LSS), and it is not uncommon for TOLF to be misdiagnosed as LSS, resulting in both delayed diagnosis and management [5, 6].

Isolated or continuous TOLF has been frequently reported in literature, but there are very few reports of co-existing thoracic and lumbar stenosis or non-continuous multilevel TOLF [7–9]. The severity of stenosis in one region may mask the symptoms of other regions, which makes the diagnosis difficult, on account of the complex clinical manifestation (combined upper motor neuron and lower motor neuron signs) [8]. Because of the variability in the level and extent of the lesion, neurological status and complex clinical manifestation, choosing the most appropriate surgical strategy for tandem spinal stenosis is often difficult and should be considered on an individual basis. The difficulties in identifying the responsible lesion and the potential for the disease to progress postoperatively indicate that no consensus regarding the management of this tandem spinal stenosis currently exists.

Although not statistically determined, surgeons and patients may prefer to choose staged decompression surgery in accordance with the location of the primary lesion that is responsible for the main clinical symptoms, and may regard single-staged decompression as too invasive. In the present study, we reviewed the surgical management and early outcome of eleven such patients with multilevel TOLF coexisting with/without LSS treated in a two-stage manner and discuss the staged surgical strategy with respect to individual focal characteristics based on retrospective analysis.

## Methods

### Patients

Between October 2007 and May 2014, consecutive 11 patients were treated with a 2-stage surgical method for multilevel TOLF coexisted with/without LSS at our institute. The minimum follow-up period was twelve months. This series included 4 males and 7 females, with a mean age at the initial surgery of 50.2 ± 11.8 years (range, 30–65 years). The duration of postoperative follow-up was 21.2 ± 12.2 months (range, 12 months – 55 months). This study was approved by the institutional review board of Peking Union Medical College Hospital. And written informed consent was obtained from all participants in the study.

### Operative indications

Retrospective review of the medical records and radiographic images was done. Criteria for prospective candidates for 2-stage decompression surgery included: (1) multilevel TOLF with/without LSS, as shown on CT and MRI of the total spine; (2) neurological deficits consistent with the radiological results; (3) a general condition allowing staged surgical interventions; and (4) high motivation for improvement in activities of daily life and a wish to undergo staged decompression.

### Operative procedures

**Fig. 1:**
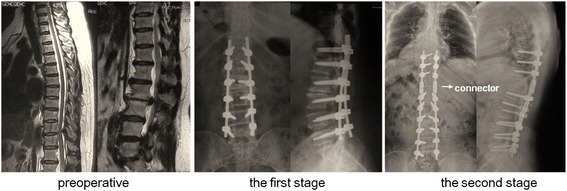
Connectors applied in the junctional area. A 54-year-old female patient with spinal stenosis in thoracic and lumbar regions underwent staged surgeries (case 7). During the first stage of surgery, we deliberately left the cranial stump of the rods approximately 2.0 cm in length. During the second stage of surgery, two series of connectors (marked by the white arrow) were applied to the junctional connection

All surgeries were performed by the same orthopedic surgical team. Posterior laminectomy with instrumentation using pedicle screws was performed for all the patients. Circumferential decompression through a transpedicular approach was performed in one patient with severe thoracic OPLL at the second stage of surgery. Pedicle screws with locally harvested bone grafts and inter-body cage were used at the lumbar region involved to provide the patient with sufficient stability. During the first stage of surgery, we deliberately left the cranial or caudal stump of the rods approximately 2.0 cm in length, in order to facilitate connecting the rods of the second stage of surgery to that of the first stage. During the second stage of surgery, two series of connectors were applied to the junctional connection. (Figure 1) For patients with dural ossification, we chose to excise the ossific dura mater with no material grafted. In addition, we used cell saver to minimize blood loss and utilized combined intraoperative transcranial electric stimulation motor evoked potential (MEP) and somatosensory-evoked potential (SEP) for all procedures.

### Surgical sequences

When deciding on the surgical sequence, the objective of the first stage of operation was to relieve the patient’s main clinical symptoms, whilst taking into the consideration the patient’s compliance. Therefore, identification of the responsible lesion and the potential for the disease to progress postoperatively may be the most important and difficult task for us to undertake before the fist procedure.

**Fig. 2:**
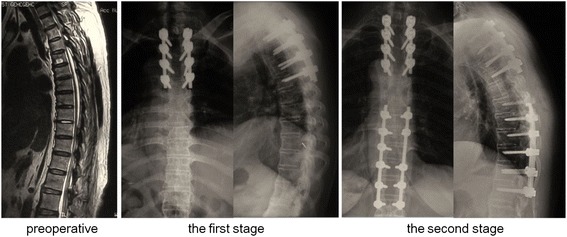
Surgical Sequences. A 61-year-old female patient with multilevel TOLF at the level of T3-T4 and T9-T12 was treated the upper lesion first, followed by the lower one

We need to identify the responsible lesion with regards to the extent and distribution of the lesion, the results electrophysiological studies, as well as the patient’s neurological status and medical comorbidities. For patients with serious radicular symptoms or cauda equina syndrome, we performed lumbar surgery first followed by thoracic surgery. For patients without radicular symptoms or cauda equina syndrome, we undertook the thoracic operation first. For patients with non-continuous multilevel TOLF and urinary dysfunction, we treated the lower lesion first, followed by the upper one (Fig. 2).

### Time interval between Two operations

All the patients returned to home while waiting for their second-stage operation. The time interval between the staged surgeries should be determined reasonably and individually, taking into account the patients’ psychological and physiological endurance to the second surgery and the recovery of neurological function. In general, we recommend that the second stage of surgery to be performed 3–6 months after the first stage surgery. However, in cases whose neurological status deteriorates sharply after fleeting signs of recovery, the second surgical intervention should be performed as soon as possible.

### Clinical outcomes

Amount of blood loss during surgery and operation time were recorded. Time for the 2-stage operative surgical time was measured respectively. The patient’s neurological status were evaluated according to modified Japanese Orthopaedic Association (mJOA) score [8], and mJOA improvement rate (expressed as %) was calculated as (postoperative JOA score - preoperative JOA score/11 - preoperative JOA score) × 100 %. The recovery of nerve function was divided into four groups according to the IR of JOA score: excellent (IR ≥ 75 %), good (75 % > IR ≥ 50 %), fair (50 % > IR ≥ 25 %), and poor (IR < 25 %). The evaluation of neurological status was done at 1 week before the first stage of surgery, at 3 months after the first stage, at 1 week before the second stage of surgery, and at the final follow-up.

Intraoperative and postoperative complications were analyzed. Complications were graded as major or minor. Major complications were those which had an effect on the final outcome. Minor complications were the one which did not have an effect on the final outcome and were managed conservatively [10].

### Statistical analysis

The data were processed using SPSS 18.0 statistical software (SPSS, Inc., Chicago, IL, USA). All quantitative data are expressed as mean ± standard deviation (SD). Paired t tests were used to compare mJOA preoperatively, 3 months after the first stage of surgery, 1 week before the second stage of surgery, and at final follow-up. Independent sample *t* test were used for the comparison of the parameters between each stage operation, including operation time, blood loss, number of operated levels and hospital stages. A *P* < 0.05 was considered statistically significant.

## Results

A summary of the demographic data of patients was illustrated in Table 1. The mean Body Mass Index (BMI) was 29.7 ± 5.5 (range, 21.6–41.0). 8 patients (72.7 %) had BMI greater than 28. The mean preoperative duration of symptoms was 24.4 ± 36.3 months (range, 2–120 months). Eight out of 11 patients (72.7 %) complained of urinary dysfunction.

**Table 1:** Clinical information of the 11 cases associated with multilevel TOLF

Case	Age(years)	Sex	Body Mass Index	Coexisting Disease	Symptoms duration (months)	Acute Exacerbation Duration (months)	Preoperative Symptoms	Urinary Dysfunction	Etiology of TSS	Number of Involved	Follow-up Period (months)
1	34	M	32.7	N	6	2	Spastic paraparesis, pyramidal signs, sensory dysfunction	N	OLF, DO	4	55
2	56	F	26.7	N	60	6	Low back and leg pain, lower limb numbness, sensory dysfunction	N	OLF	5	17
3	65	F	29.3	Y	120	2	lower limb numbness, spastic paraparesis, sensory dysfunction, sphincter dysfunction, pyramidal signs	Y	OLF, OPLL, DO	7	14
4	49	F	21.6	N	6	1	lower limb numbness, sensory dysfunction, spastic paraparesis, pyramidal signs	Y	OLF, OPLL, DO	8	23
5	58	F	29.7	Y	120	12	low back and leg pain, lower limb numbness, sensory dysfunction, pyramidal signs, sphincter dysfunction	Y	OLF, OPLL	9	21
6	30	M	35.1	N	6	3	lower limb numbness, sensory dysfunction, spastic paraparesis, pyramidal signs, sphincter dysfunction	Y	OLF, OPLL	9	14
7	54	F	30	N	6	3	low back and leg pain, lower limb numbness, intermittent claudication	N	OLF	4	18
8	61	F	29.6	N	13	9	low back pain, lower limb numbness, sensory dysfunction, spastic paraparesis, pyramidal signs, sphincter dysfunction	Y	OLF, OPLL, DO	4	18
9	56	F	29.3	N	2	2	lower limb numbness, sensory dysfunction, pyramidal signs, sphincter dysfunction	Y	OLF, OPLL DO	9	13
10	54	M	21.9	N	36	0.3	lower limb numbness, sensory dysfunction, spastic paraparesis, pyramidal signs, sphincter dysfunction	Y	OLF, DO	13	28
11	35	M	41	Y	3	3	lower limb numbness, sensory dysfunction, pyramidal signs, sphincter dysfunction	Y	OLF, OPLL	5	12
Average	50.2		29.7		24.4	4.2				7	21.2
SD	11.8		5.5		36.3	4.1				2.9	12.2

Regarding the etiology of TSS, two cases only had TOLF, two cases had TOLF with dural ossification (DO), three cases had TOLF with thoracic ossification of the posterior longitudinal ligament (OPLL), and the remaining four cases had TOLF with thoracic OPLL and DO.

**Fig. 3:**
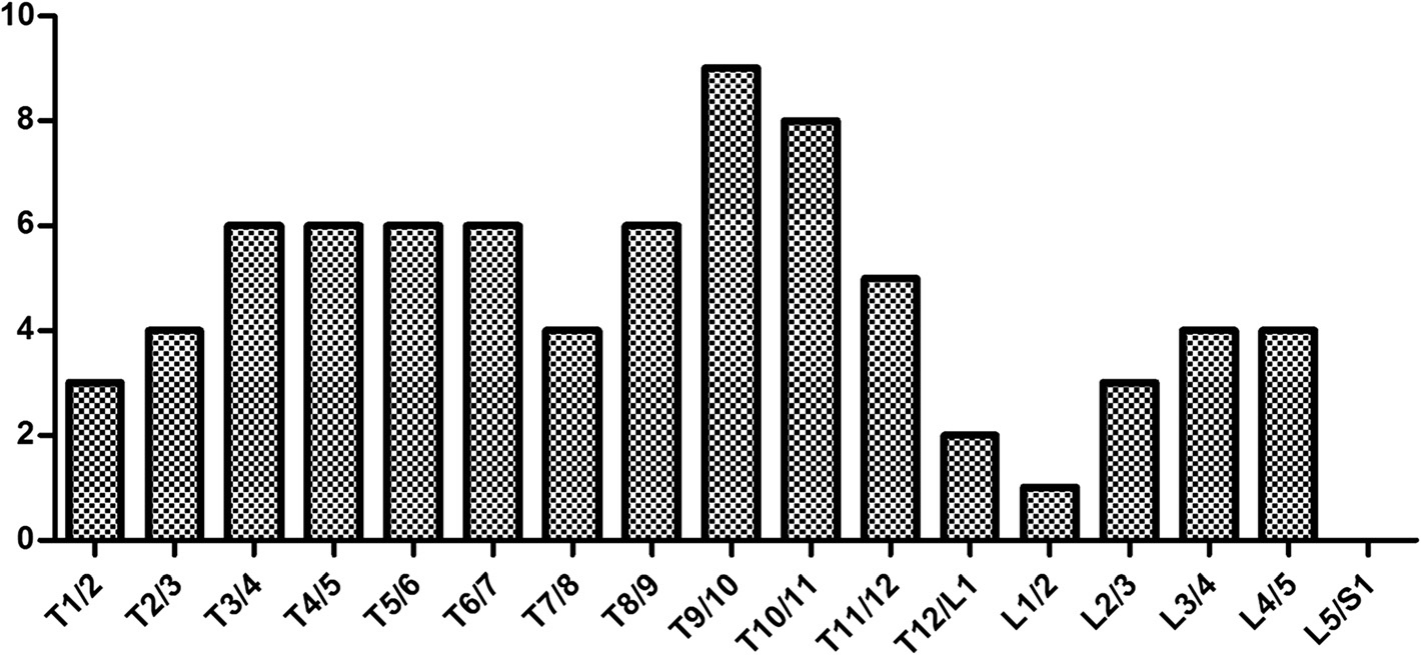
Distribution of the OLF in relation to the intervertebral disc level

The compression levels affecting the spinal cord considered to be responsible for the thoracic myelopathy were shown in Fig. 3**.** Of the 77 affected intervertebral disc levels, we observed spinal stenosis at the lower thoracic segments (T9-T10, T10-T11, T11-T12, and T12-L1) in 31.2 % of the affected intervertebral disc levels, at the middle thoracic segments (T5-T6, T6-T7, T7-T8, and T8-T9) in 28.6 %, at the upper thoracic segments (T1-T2, T2-T3, T3-T4, and T4-T5) in 24.7 %, and at the lumbar segments in 15.5 %.

### Operative data

In four cases, the patient underwent first stage surgery for the upper lesions (36.4 %), and the other 7 cases (63.6 %) the patient underwent first stage surgery for the lower lesions. The mean time interval between two operations was 11.1 ± 12.6 months (range, 0.5-37 months) (Table 2). In case №10, post-operative CT scans indicated mal-positioning of screws on the left side of vertebrae T8 and T9, a second surgery was performed only 15 days after the first surgery, in which the mal-positioned screws were removed and the upper lesions (T3-T7) were surgically corrected. We excluded the data of case №10 when comparing the hospital stays and the change in mJOA scores between the staged surgeries.

**Table 2:** Summary of operative data obtained in the 11 patients of multilevel TOLF

Surgical Sequence		Time Interval between Two Operations	Operated Levels		Number of Operated Levels		Operation Time (min)		Operation Time per Level (min)		Blood Loss (ml)		Blood Loss per Level (ml)		Hospital Stays (days)		Complications	
			1^st^ Stage	2^nd^ Stage	1^st^ Stage	2^nd^ Stage	1^st^ Stage	2^nd^ Stage	1^st^ Stage	2^nd^ Stage	1^st^ Stage	2^nd^ Stage	1^st^ Stage	2^nd^ Stage	1st Stage	2^nd^ Stage	1^st^ Stage	2^nd^ Stage
1	LL	34	T9-T11	T2-T4	3	3	280	270	93.3	90	600	1600	200	533	35	19	N	N
2	UL	6	T8-T11	L3-L5	4	3	175	115	43.8	38.3	500	250	125	83	15	13	N	N
3	UL	1	T1-T3	T4-T9	3	6	150	234	50	39	400	800	133	133	10	25	N	N
4	LL	37	T4-T11	T1-T2	8	2	400	245	50	122.5	1200	1500	150	750	23	19	N	CSF leakage
5	LL	6	L2-L5	T4-T10	4	7	140	230	35	32.9	700	1200	175	171	23	18	CSF leakage	CSF leakage
6	LL	5	T5-T12	T2-T4	8	3	310	205	38.9	68.3	1500	500	188	167	14	15	CSF leakage	CSF leakage
7	LL	5	L2-L5	T9-T10	4	2	230	175	57.5	87.5	800	800	200	400	15	22	N	N
8	UL	5	T3-T4	T9-T12	2	4	295	235	147.5	58.8	2000	1000	1000	250	13	26	CSF leakage	CSF leakage
9	UL	8	T1-T7	T9-T12	7	4	425	240	60.7	60	900	600	129	150	67	27	Acute epidural hematoma,wound infection	CSF leakage
10	LL	0.5	T8-L5	T3-T7	10	5	400	425	40	85	600	1000	60	200	40	_	CSF leakage, screw malposition	CSF leakage
11	LL	15	T10-L1	T3-T5	4	3	210	335	52.5	111.7	4000	2500	1000	833	8	14	N	N
Average	_	11.1	_	_	5.2	3.8	274	246	60.8	72.2	1200	1068	305.5	334	22.3	19.8	_	_
SD	_	12.6	_	_	2.6	1.6	103	81	32.8	29.9	1044	625	345.8	261	17.6	5.1	_	_
P value	_	_	_		0.154		0.488		0.407		0.723		0.831		0.371		_	

***UL*** upper lesion first, ***LL*** lower lesion first

The mean amount of blood loss was 1200 ± 1044 ml (range, 400–4000 ml) for the first stage of surgery, and 1068 ± 625 ml (range, 250–2500 ml) for the second stage. The mean operation times of the first and second staged procedures were 274.1 ± 102.7 min (range, 140–425 min) and 246.3 ± 80.6 min (range, 115–425 min), respectively. The mean amount of blood loss and operation time for each lamina were 305 ± 346 ml (range, 60 to 1000 ml) and 60.8 ± 32.8 min (range, 35.0-147.5 min) for the first stage, and 334 ± 261 ml (range,83 to 833 ml) and 72.2 ± 29.9 min (range, 32.9-122.5 min) for the second stage. The mean number of laminae resected was 5.2 ± 2.6 (range, 2–10) and 3.8 ± 1.6 (range, 2–7) for the first and second staged surgeries respectively. There were no statistically significant differences between the first and second staged surgeries, in terms of total operative blood loss, total operation time, blood loss for each lamina, operative time for each lamina, and number of laminae resected (Table 2).

### Neurologic status

**Fig. 4:**
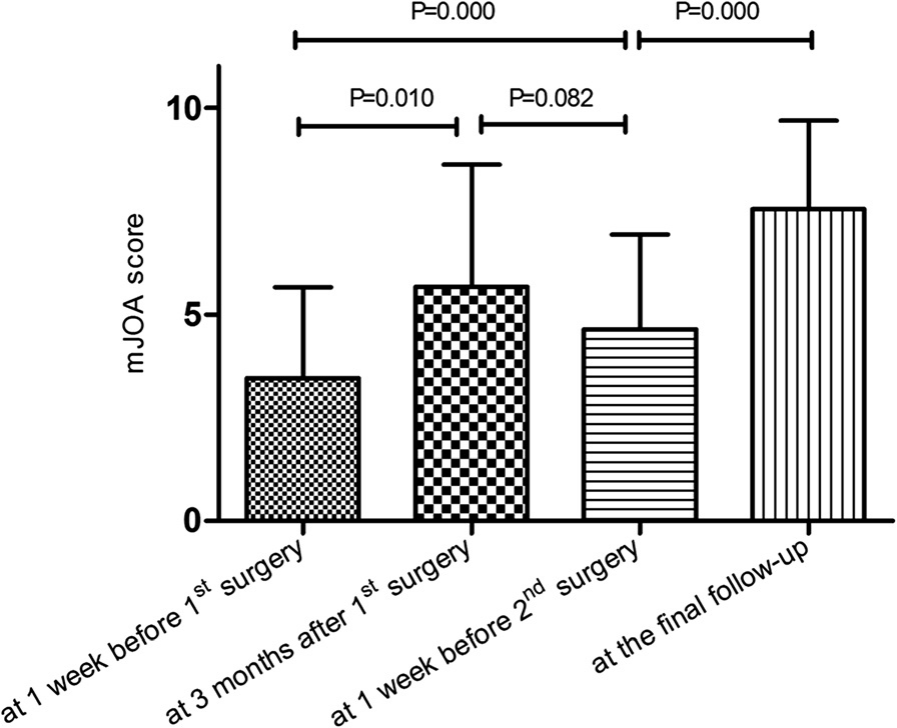
modified Japanese Orthopaedic Association (JOA) score

The preoperative mean ± SD mJOA score before the first stage of surgery was 3.5 ± 2.2 points. At the final follow up, the postoperative mean ± SD mJOA score was 7.5 ± 2.2 points. Compared with the preoperative score, the mJOA score was significantly higher 3 months after the first stage of surgery (*P* = 0.01). There was no statistically significant difference in mJOA scores at 3 months after the first stage of surgery and 1 week before the second stage of surgery, even though the average mJOA score was slightly lower at 1 week before the second stage. Statistically significant differences were observed for mJOA score (*P* = 0.00) at 1 week before the second stage of surgery, when compared with preoperative mJOA score before the first stage of surgery. The mJOA outcome improved significantly at final follow-up (P = 0.00) at an average of 21.2 ± 12.2 months, compared with mJOA score at 1 week before the second stage of surgery. The average mJOA improvement ratio was 58.2 ± 20.1 % at final follow-up (Table 3, Fig. 4).

**Table 3:** modified Japanese Orthopaedic Association (JOA) score

	mJOA				mJOA Improvement Rate at the final follow-up
Case	1 week before 1st surgery	3 months after 1st surgery	before 2nd operation	at the final follow-up	
1	4	10	6	10	85.7 %
2	6	—	7	9	60.0 %
3	2	2	2	5	50.0 %
4	3	6	5	7.5	56.3 %
5	4	6	6	7.5	50.0 %
6	0	2	2	5	45.5 %
7	7	8	8	10	75.0 %
8	2	5	5	7	55.6 %
9	3	3	3	4	12.5 %
10	1	—	1	8	70.0 %
11	6	9	6	10	80.0 %
Average	3.5	5.5	4.6	7.5	58.2 %
SD	2.2	3.1	2.3	2.2	20.1 %

### Complications

One patient (9.1 %) developed an epidural hematoma and one patient (9.1 %) was found to have screw malposition after the first stage of surgery, both of which required additional surgical intervention. Deep wound infection occurred perioperatively in the patient above-mentioned who underwent an additional surgery for epidural hematoma. The implants were successfully retained without recurrence of infection after surgical debridement and application of a vacuum drain with postoperative intravenous antibiotics. In addition, the aforesaid two patients recovered with no residual neurological deficits after emergency surgery and conservative treatment.

Intraoperative and postoperative, cerebrospinal fluid (CSF) leakage was observed in 4 cases (36.4 %) after the first stage operation and in 6 cases (54.5 %) after the second stage operation. Most of them found to have dural ossification preoperatively (Table 2).

There were no medical complications that could directly be related to the staging of posterior stabilization surgery. Furthermore, no major peri- or post-operative medical complications or major bleeding complications occurred (e.g. myocardial infarction, pneumonia, pulmonary embolus, adult respiratory distress syndrome, disseminated intravascular coagulation, or death). The occurrence of a complication itself did not affect patients’ recovery of the neurologic function, but did increase the length of hospital stay.

## Discussion

Although the surgical treatment and outcomes of a limited number of multilevel TOLF patients have been reported in the literature before [5, 7–9, 11], to the best of our knowledge, this study is the first to report that a staged surgical strategy for non-continuous multilevel TOLF can be practicable, safe and provide satisfactory clinical outcomes.

Concomitant noncontiguous level TOLF is a relatively uncommon finding that produces myelopathy in the thoracic area. Most of the patients showed the typical features of thoracic myelopathy, including sensory and motor deficits in the trunk and lower extremities, sphincter disturbance, and pyramidal signs, as found in our series. Coexistent lumbar stenosis accounts for lower motor neuron deficits and may mask long tract signs due to compression in the L4-S2 (epiconus) region [6, 12]. There have been a few reports describing the clinical course of tandem thoracic with/without lumbar stenosis [8, 9].

For patients with ossification of the dura matter detected preoperatively, this could increase the technical difficulty of surgical decompression, the ossified ligament should be removed carefully in order to avoid dural laceration and iatrogenic spinal cord injury. In our series, dural ossification was detected in 6 out of 11 patients by means of radiological investigation preoperatively, and confirmed during surgery. In order to achieve complete decompression, we had to dissect the ossified dura matter. Thus, four of those patients experienced CSF leak postoperatively.

Surgical management of multilevel thoracic ossification of ligamentum flavum, especially discontinuous multi-segmental lesions, can be a complex undertaking involving considerable risk. Two surgical strategies have been proposed: single-stage surgery or two-stage surgery. Eskander et al. showed that simultaneous decompression of both the cervical and lumbar spine in TSS may reduce the chance of infection, minimize hospital stay and related costs, and maximize patient function recovery [13]. However, when considering the invasive nature and increased incidence of complications, we believe that the strategy of dividing the surgery into two smaller, staged posterior procedures is a safer option and reduces the need for additional corrective surgery should complications occur.

There are a number of potential advantages to two-stage posterior surgery in these complex patients. Firstly, this two-stage surgery may result in better neurological functional recovery than single-stage surgery or selective decompression. Matsumotoc et al. [8] reported that, 18 patients were treated with a single-stage surgical method or a selective decompressive surgical method for TOLF coexisted with LSS. The average JOA improvement rate was 17.3 % postoperatively, which was lower than 58.2 % in the present study. In a study by Gupta et al. [9], 82 % of the patients with concomitant noncontiguous thoracic and lumbar stenosis had excellent or good clinical results at the last examination. According to their surgical outcome criteria, all the patients in the present study had excellent or good clinical results at the final follow-up. Secondly, operative and anesthetic time of each individual staged operation is much shorter than that of single-stage surgery. Thus, the risk of hemodynamic disorder and pulmonary complications is lessened in staged surgery. In our series, no patient sustained a major perioperative cardiopulmonary complication or death. Thirdly, in two-stage surgery, each individual stage is less surgically invasive and a single-stage operation and therefore intensive care unit (ICU) stays are unnecessary in most patients, reducing the risk for iatrogenic complications such as infectious complications. In our series, only two patients who sustained large blood loss required any postoperative ICU stays. In addition, shorter procedures lead to reduced surgeon fatigue and fewer changes of the anesthetic and nursing teams during the operation. This may be a factor in reducing the risk of iatrogenic injuries or complications.

Nevertheless, there are still some potential drawbacks to staged surgery. Firstly, the combined anesthetic time is longer for two-stage than single-stage surgery, because more time is required for two anesthetic setups and recovery. However, we encountered no complications from repeated general anesthesia in the patients in our study. Secondly, there is a potentially a higher risk of wound complications, such as infections and dehiscence. Only one patient in our series sustained a deep wound infection (9.1 %). Because of the absence of a suitable control group, it is unclear whether the patients in our series would have had a higher deep infection rate with two posterior operations compared to one. In addition, if a major medical complication occurs during the first stage of surgery, this patient may be medically unfit to undergo the second stage of surgery, hence making it difficult for a direct comparison with a suitable control group. Although possible, there were no major medical complications in present study, and as a result, all patients were able to complete the staged surgeries as planned. In addition, staged surgery means staged neurological functional recovery. Thus, there is a concern that neurological deficits may deteriorate during the time interval between the two staged operations. The results of our current study also revealed this phenomenon by decreased mJOA before the second stage of surgery compared with that after the first stage of surgery. In our view, time interval between staged operations and treatment compliance in these patients should be emphasized. Taking into consideration the patients’ physiological and psychological endurance, we recommend that the second operation to be performed 3–6 months after the first stage of surgery. However, in cases where the neurological status deteriorates sharply after fleeting signs of recovery, the second surgical intervention is considered to be undertaken as soon as possible.

In cases with symptomatic multilevel spinal stenosis in thoracic and lumbar region, staged procedures have the dilemma regarding the choice of the region to be surgically addressed first. Currently, there’s no report in the existing literature that clearly indicates a preference to surgical sequence. In our view, the decision at the first posterior operation should be based predominantly on the location of the responsible compressive pathology within the spinal canal. For patients with severe radicular symptoms or a definitive diagnosis of cauda equine syndrome, the lumbar lesions should be operated on first. For other cases, in our opinion, thoracic lesions or upper lesions should be handled first. The aforementioned recommendation is mainly based on the following considerations. Firstly, the lower tracts (i.e. lumbar) that pass through the upper region (i.e. thoracic) get decompressed thus addressing the thoracic region and thereby improving lumbar symptoms [10, 14]. Secondly, the duration of symptoms has a negative effect on the clinical outcome of thoracic decompression [8, 15]. Thus the earlier we operate on the TSS, the better clinical outcomes we will achieve.

Patients in the current series generally experienced significant improvements in neurological function and activities according to the mJOA score after operation. A total of 81.8 % (9/11) patients had excellent or good clinical outcomes according to the improvement rate (IR) of mJOA score, and the improvement rate was 58.2 ± 20.1 %,in line with previous literature [1]. The results of this study showed that staged combined thoracic and lumbar decompression provide a comparable clinical outcome to other reported surgical procedures for multilevel spinal stenosis [8, 10, 13, 16]. However, the phenomenon of late deterioration after initial postoperative improvement was also recognized in this present study. The mJOA outcome achieved at 1 week before the second stage of surgery was found to be lower than that at 3 months after the first stage of surgery, however the difference was not statistically significant (*P* = 0.082). In selected patients, late deterioration because of other untreated lesions is not uncommon [5, 10]. Fushimi et al. [5] described six patients who suffered unexpected acute neurological deterioration after lumbar decompression surgery due to recurrent stenosis at the thoracic spine. The cause of neurological deterioration under these circumstances is not known, although several hypotheses, including change of pressure at the level of missed compressive lesions [17], postoperative change of dynamics of the flow of cerebrospinal fluid [18], spinal cord infarction and cord edema [19], have been proposed. However, further long-term outcome still remains to be seen. In clinical practice, it is important for surgeons to warn patients of the risk of progression of neurologic deficit following single-stage surgery [20].

There were some limitations of this study. The main limitation of the study was the small sample size as multilevel TOLF was a clinically rare disease. According to the literature, only one case report reported a patient with multilevel spinal canal stenosis of the thoracolumbar spine underwent two-stage decompression [12]. Besides, the present study can only provide early clinical outcomes due to a relatively short follow-up. A longer-term follow-up of a prospective cohort is needed in the future to confirm the present results and evaluate the selection criteria. Another limitation was that no comparative cohort studies have yet been performed with patients treated by single-stage surgery. Future studies should prospectively compare clinical outcomes of staged surgery with those treated with single discontinuous multilevel decompression. In addition, no cost-analysis was performed with regards to the additional hospital stay in two-stage surgery compared to single stay surgery.

## Conclusions

In conclusion, this study indicated that the staged surgical treatment for multilevel TOLF was a viable surgical option with acceptable complication rates and good clinical outcomes. However, the surgeon needs to be acutely aware of a potential higher risk of neurological deterioration after the first stage of surgery, and clear and comprehensive preoperative communication the patient is essential for informed consent. Further accumulation of experience is essential to justify indication of our two-staged procedure for cases with discontinuous multilevel spinal stenosis in lumbar and thoracic regions.

## Abbreviations

TOLF: Thoracic ossification of ligamentum flavum
mJOA: Modified Japanese Orthopedic Association score
TSS: Thoracic spinal stenosis
OLF: Ossification of ligamentum flavum
CT: Computed tomography
MRI: Magnetic resonance imaging
LSS: Lumbar spinal stenosis
MEP: Motor evoked potential
SEP: Somatosensory evoked potential
IR: Improvement rate
BMI: Body mass index
DO: Dural ossification
OPLL: Ossification of the posterior longitudinal ligament
CSF: Cerebrospinal fluid
ICU: Intensive care unit
